# Supplementary material for: Immunosuppression of Tumor-Derived Factors Modulated Neutrophils in Upper Tract Urothelial Carcinoma Through Upregulation of Arginase-1 via ApoA1-STAT3 Axis
Source: Cells. 2025 Apr 30;14(9):660. doi: 10.3390/cells14090660 (PMC12072159; doi:10.3390/cells14090660)
Supplement: Supplementary file 1 [file cells-14-00660-s001.zip › cells-3601956-supplementary.pdf]

Supplementary Information

Neutrophil treated Adiponectin and quantitative real-time PCR

Neutrophils isolated from whole blood of donors were treated with 10μg/ml adiponectin (Enzo Life Science) or control medium in the presence of 5% CO_2, at 37°C. One hour later, neutrophils were washed by 1x PBS twice and dissolved in Trizol reagent for total RNA isolation. Quantitative real-time PCR with a SYBR Green on StepOne cycler (Applied Biosystems/Life Technologies) was used for amplification of genes. Specific primers for *ARG1* gene included F: 5′-GGCAAGGTGATGGAAGAAAC-3′ and R: 5′-AGTCCGAAACAAGCCAAGGT-3′; and for *ACTB* gene included F: 5′-TGCGTGACATTAAGGAGAAG-3′ and R: 5′-GCTCGTAGCTCTTCTCCA -3′. Expression of target gene was determined relative to that of β-actin, and the relative fold change was calculated by the ΔΔCt method.

Western blot

Neutrophils were rinsed in cold PBS and the total protein were obtained using M-PER^{TM} mammalian protein extraction reagent (Thermo Fisher). Protein samples or culture supernatant of tumor tissue (TTCS) were applied to SDS-PAGE and transferred onto the PVDF membrane. After blocking with 5% skimmed milk at room temperature for 1 h, the membranes were incubated overnight with primary antibodies at 4 °C and subsequently washed by Tris-buffered saline within tween 20. The horseradish peroxidase-conjugated goat anti-rabbit secondary antibody was incubated with the membranes at room temperature for 1 h. The MultiGel-21 imaging system (TOPBIO) was employed to visualize the protein signals. The measurement of relative optical density was determined using Image J software. The primary

antibodies were: apolipoprotein A1 (1:1000, #14427-1-AP; Proteintech) and arginase-1 (1:1000, #16001-1-AP; Proteintech).

Supplementary Figure legends

Fig. S1 Gating strategy for the analysis of neutrophils by flow cytometry**.**

After removal of erythrocytes, whole blood cells (left panel) or isolated neutrophils (right panel) were stained with fluorescent dye conjugated Abs and followed analyzed by flow cytometer. Isolated neutrophils displayed pure (99.3%) CD66b+CD11b+ population (right panel).

Fig. S2 Western blot analysis of apolipoprotein A1. Apolipoprotein A1 (Apo-A1) protein in culture supernatant of tumor tissue (TTCS) from 3 patients with UTUC (#1, #2 and #3, 1:1000 dilution, middle lanes) or control medium (ctrl, left lane) were detected by western blot. The serum sample (serum, 1:1000 dilution, right lane) was used as positive control.

Fig. S3 Adiponectin did not increase RNA level of arginase-1 in neutrophils.

Primary neutrophils were treated with 10μg/ml Adiponectin or control medium for 1hrs. And total RNA were extracted and followed used for quantitative PCR. The statistical analysis of RNA level of *ARG1* relating to internal control were represented.

Fig. S4 Western blot analysis of arginase-1 in neutrophils. **A** Neutrophils were preincubated with a combination of anti-SRB1 and anti-ABCA1 antibodies (blockers) for 30 minutes. Subsequently, cells were treated with TTCS or Apo-A1 or control medium (ctrl) and the protein expression of arginase-1 was detected by western blot.

Histone H3 was used as loading control. Lanes from left to right: marker, ctrl, TTCS+blockers, Apo-A1+blockers, TTCS and Apo-A1. **B** The statistical results for arginase-1 levels relative to loading control (Arg-1/H3) are presented. Two independent experiments were performed. #$p < 0.05$ and ##$p < 0.01$ compared with ctrl group; *$p < 0.05$, and **$p < 0.01$, unpaired t-tests.

Figure S1.

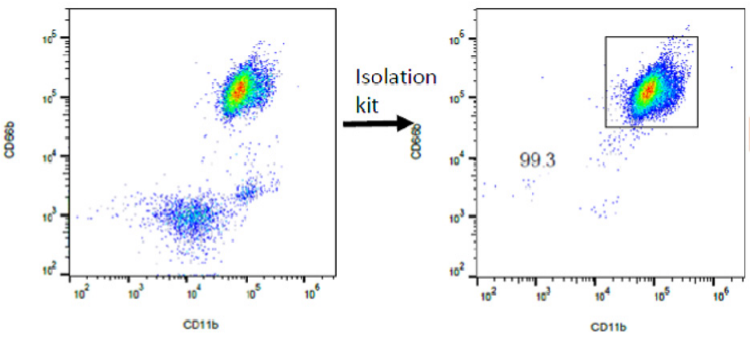

Figure S2.

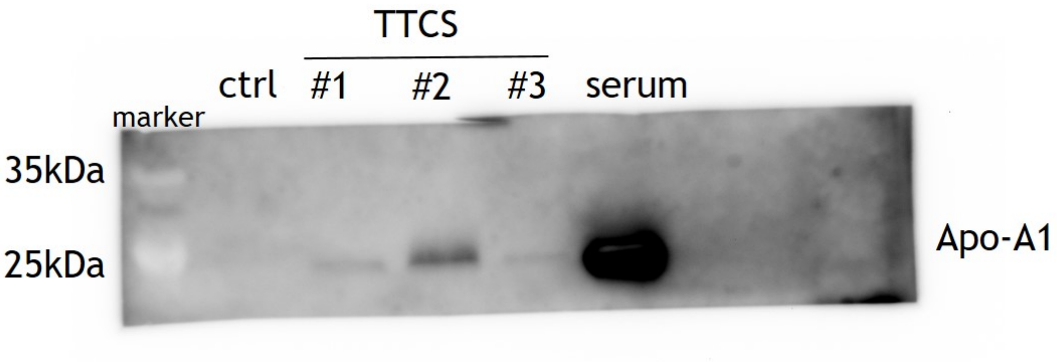

Figure S3.

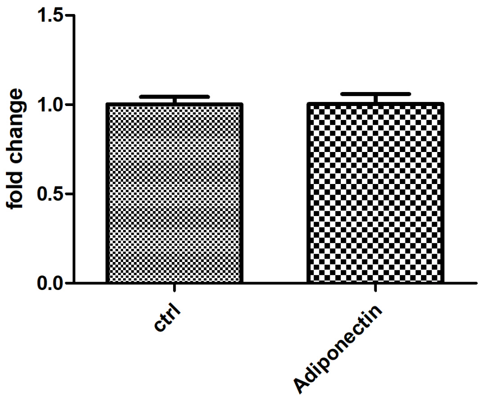

Figure S4.

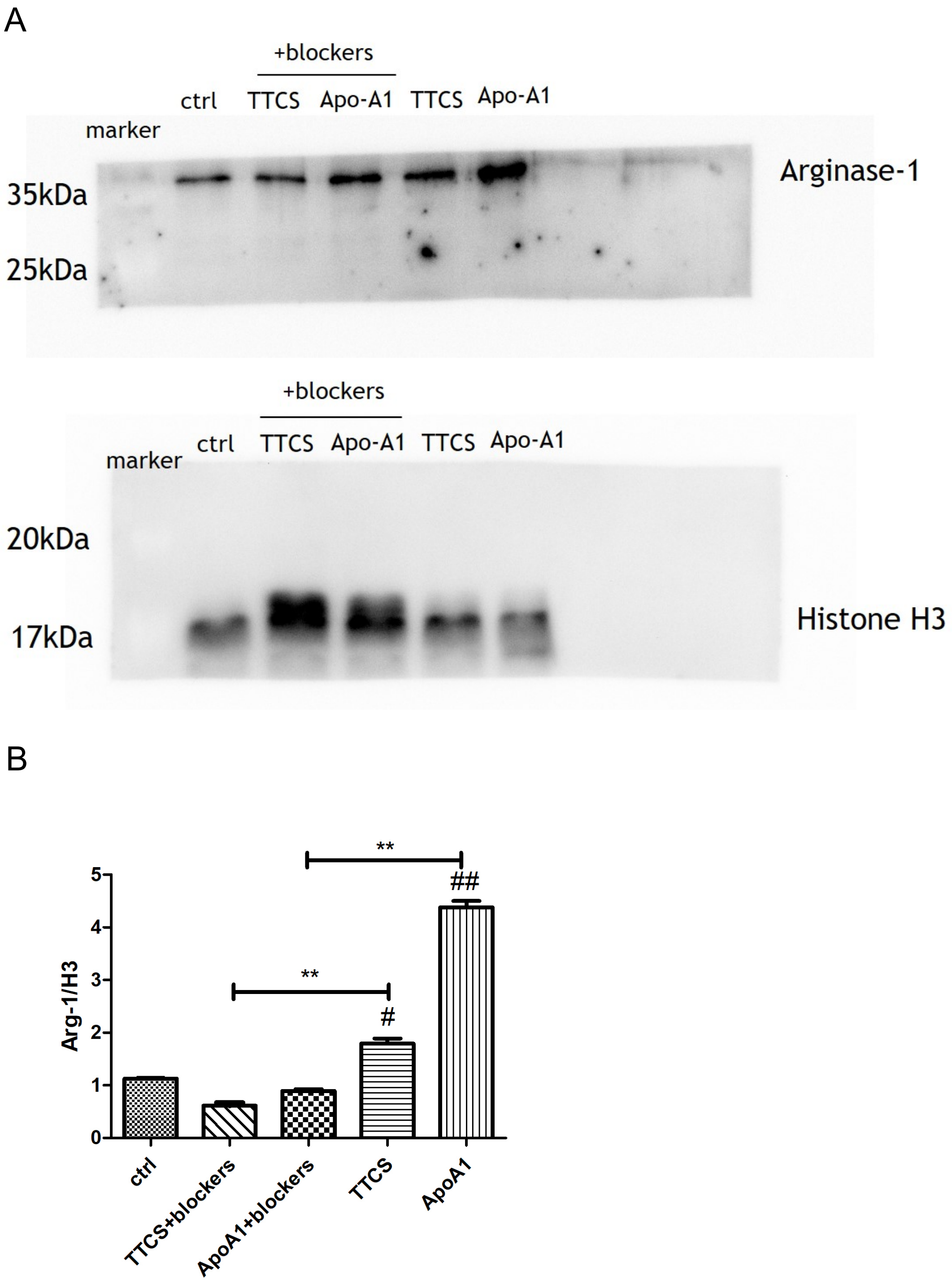
A
+blockers
ctrl
TTCS
Apo-A1
TTCS
Apo-A1
marker
35kDa
25kDa
Arginase-1
+blockers
ctrl
TTCS
Apo-A1
TTCS
Apo-A1
marker
20kDa
17kDa
Histone H3
B
Arg-1/H3
0
1
2
3
4
5
**
**
#
##
ctrl
TTCS+blockers
ApoA1+blockers
TTCS
ApoA1
